# Solar UV exposure among outdoor workers in Denmark measured with personal UV-B dosimeters: technical and practical feasibility

**DOI:** 10.1186/s12938-017-0410-3

**Published:** 2017-10-10

**Authors:** Kasper Grandahl, Ole Steen Mortensen, David Zim Sherman, Brian Køster, Paul-Anker Lund, Kristina Sophie Ibler, Paul Eriksen

**Affiliations:** 1 The Department of Occupational Medicine, Copenhagen University Holbæk, Gl. Ringstedvej 4B, 4300 Holbæk, Denmark; 2 Section of Social Medicine, Department of Public Health, University of Copenhagen, Øster Farimagsgade 5, 1014 Copenhagen, Denmark; 3 Scienterra Limited, Oamaru, New Zealand; 4 The Danish Cancer Association, Copenhagen, Denmark; 5 National Research Centre for the Working Environment, Copenhagen, Denmark; 6 The Department of Dermatology, Copenhagen University Roskilde, Copenhagen, Denmark; 7 The Danish Meteorological Institute, Copenhagen, Denmark

**Keywords:** Ultraviolet exposure, UV-B dosimetry, Occupational skin cancer, Danish outdoor workers, Technical and practical feasibility

## Abstract

**Background:**

Exposure to solar ultraviolet radiation is a well-known cause of skin cancer. This is problematic for outdoor workers. In Denmark alone, occupational skin cancer poses a significant health and safety risk for around 400,000 outdoor workers. Objective measures of solar ultraviolet radiation exposure are needed to help resolve this problem. This can be done using personal ultraviolet radiation dosimeters.

**Methods:**

We consider technical and practical feasibility of measuring individual solar ultraviolet exposure at work and leisure in professions with different á priori temporal high-level outdoor worktime, using aluminium gallium nitride (AlGaN) photodiode detector based personal UV-B dosimeters. Essential technical specifications including the spectral and angular responsivity of the dosimeters are described and pre-campaign dosimeter calibration applicability is verified. The scale and conduct of dosimeter deployment and campaign in-field measurements including failures and shortcomings affecting overall data collection are presented.

**Results:**

Nationwide measurements for more than three hundred and fifty workers from several different professions were collected in the summer of 2016. On average, each worker’s exposure was measured for a 2-week period, which included both work and leisure. Data samples of exposure at work during a Midsummer day show differences across professions. A construction worker received high-level occupational UV exposure most of the working day, except during lunch hour, accumulating to 5.1 SED. A postal service worker was exposed intermittently around noon and in the afternoon, preceded by no exposure forenoon when packing mail, accumulating to 1.6 SED. A crane fitter was exposed only during lunch hour, accumulating to 0.7 SED. These findings are in line with our specialist knowledge as occupational physicians.

**Conclusions:**

Large-scale use of personal UV-B dosimeters for measurement of solar ultraviolet radiation exposure at work and leisure in Denmark is indeed feasible from a technical and practical viewpoint. Samples of exposure data shown support the presumption that the Danish campaign UV-B dosimeter measurement dataset can be used to sum and compare exposure between groups of professions with reliable results to be used in future analysis with clinical as well as epidemiological/questionnaire data. This was despite some dosimeter failures and shortcomings.

## Background

### Exposure to solar UV among outdoor workers in Denmark

Ultraviolet radiation (UV) is a WHO (World Health Organization) group 1 carcinogen, and solar UV is the main cause of skin cancer. Outdoor workers’ exposure to solar UV occurs repeatedly on most weekdays in the summer season. We believe, as an occupational health problem this has been neglected too long. International dermatology experts have recently raised this issue and call for objective exposure measurements during working hours [1, 2].

In Denmark, there are around 400,000 outdoor workers at risk of occupational skin cancer. To assess the scale of UV exposure for Danish outdoor workers our objective was to obtain reliable measurements of individual occupational UV exposure for several hundred outdoor workers of different professions in 2016 and 2017.

In cooperation with the Danish Cancer Society, we chose to use the accessible and affordable electronic UV-B dosimeters developed by Scienterra Limited and used in Denmark in a previous study [3]. We considered using the SunSaver dosimeter or the GENESIS-UV dosimeter, both of which measures in the UV-B spectrum (280–315 nm) and in the UV-A spectrum (315–400 nm). In comparison, the Scienterra dosimeter only measures UV-B. The GENESIS-UV dosimeters is furthermore equipped with an accelerometer. However, both alternatives were inaccessible at the time and significantly more costly [4, 5].

The aim of this study is to describe the method, including the feasibility, of measuring personal UV radiation exposure using electronic UV-B dosimeters, from a technical and practical viewpoint.

In conclusion, samples of campaign exposure data for a construction worker, a postal worker and a crane fitter at work are presented and discussed.

## Methods

### Electronic UV-B dosimeter

The dosimeter measures 36 mm in diameter with a thickness of 12 mm and a weight of 26 g. A docking cradle is available from Scienterra that, together with a terminal communication program (e.g. HyperTerminal, Tera Term, Putty or the like) is used to connect to and configure the dosimeter. Powered by a lithium coin cell battery (CR1632) the dosimeter consists of an aluminum gallium nitride (AlGaN) photodiode detector [6] that has a very low sensitivity to radiation with wavelengths above approximately 320 nm (insensitive to light), a microcontroller, an (switched) integrator with a 12-bit analogue-to-digital converter (ADC) and memory to store about one million measurements. The microcontroller continually converts UV radiation measured by the detector to a digital number (integer between 0 and 4095) and stores each measurement in memory together with a timestamp.

### Dosimeter integration time

The photodiode detector signal current charges a capacitor and after a short (integration) time the capacitor voltage is sampled by the ADC and converted to a digital value (0–4095) which then represents the irradiance measured by the dosimeter. Subsequently, the capacitor is quickly discharged and the cycle repeats. To obtain a workable range of digital values the integration time can be adjusted but care must be taken not to overload or saturate the dosimeter. Longer integration times increase the dosimeter sensitivity but there is a risk of saturating the dosimeter if it is exposed to intense UV. During calibration of a batch of dosimeters, each dosimeter should have its integration time adjusted so that all dosimeters record similar digital values during calibration. However, when adjusting the integration time it must be taken into account what the maximum expected irradiance is during any following field campaign. This is handled in the dosimeter configuration by entering a “desired UV” value that equals the maximum level of UV radiation, as per the UV index (UVI), of the reference instrument during calibration divided by the maximum UVI expected during the planned field campaign. An algorithm in the microcontroller then adjusts the integration time to cover the expected UVI range automatically [7].

### Dosimeter spectral and angular responsivity

The International Commission on Illumination (CIE) action spectrum [8] is used to calculate the erythemally effective UV irradiance for any source whose spectrum is known or measured. Technically, the erythemally effective UV irradiance—often called the CIE-weighted irradiance—equals the integral of the product of the source spectral irradiance and the CIE action spectrum algebraic expressed as ${\text{E}}_{{{\text{ery }}({\text{CIE}} - {\text{weighted irradiance}})}} = \, \sum {\text{ E}}_{{\lambda \, \left( {\text{source spectral irradiance}} \right)}} * {\text{ S}}_{{{\text{ery }}({\text{CIE action spectrum}})}} \left( \lambda \right){\text{ d }}\left( \lambda \right)$.

**Fig. 1:**
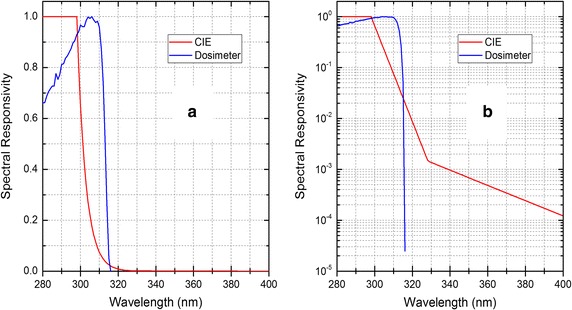
CIE or erythema action spectrum (red) and dosimeter spectral responsivity (blue) measured for an earlier version of the dosimeter. **a** Linear scale Y-axis. **b** Logarithmic scale Y-axis

The unit is (erythema-effective-) W/m^2^, not to be confused with the total or unweighted UV irradiance in W/m^2^ since the prefix “erythema-effective” is mostly omitted. The value of the CIE action spectrum is one (1.0) for wavelengths between 250 and 298 nm—indicating the most harmful radiation—and with steeply decreasing values (power law) with increasing wavelength until 328 nm where its value is 0.0015, followed by a moderate decline (power law) with increasing wavelength until 400 nm where its value is 0.00012 (Fig. 1). Here best illustrated on a logarithmic scale (Fig. 1b). The dosimeter’s spectral responsivity (Fig. 1) is almost entirely in the UV-B (280–315 nm) with hardly any response in the UV-A (315–400 nm) [3] and it does not mimic the CIE action spectrum very well. This means for example that a horizontally placed dosimeter and a small solar elevation will measure a very small signal, if any, because the major portion of the CIE-weighted irradiance lies above 320 nm, the UV-A contribution to the CIE-weighted irradiance is important for low sun. However, for high solar elevations the contribution of UV-A radiation to the CIE-weighted irradiance does not matter so much because most of the CIE-weighted irradiance is below 320 nm. This illustrates the fact that the solar position relative to the input surface of the dosimeter (aka solar elevation), the solar spectrum and the detector’s spectral responsivity are all important for calculating the radiation measured by the detector. Since the solar spectrum for any given solar elevation depends on the ozone layer thickness, the latter also affects the detector output.

**Fig. 2:**
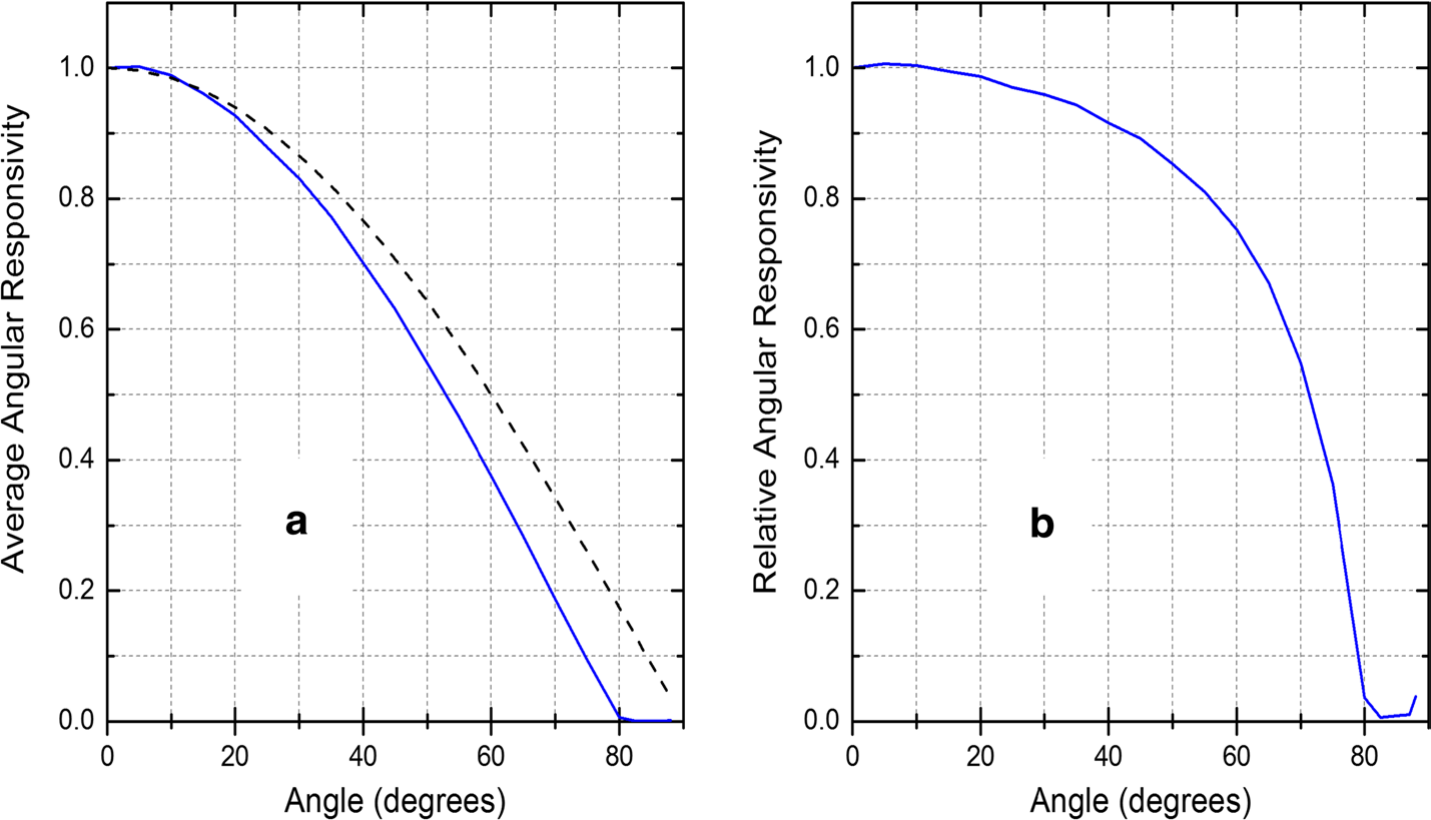
Angular responsivity for an earlier version of the dosimeters. **a** Average angular responsivity of the dosimeters (blue) and the ideal cosine (dashed). **b** Relative angular responsivity of the dosimeters (blue) to the ideal cosine

The angular responsivity (Fig. 2) of an earlier version of the dosimeter [3] is quite good for angles of incidence (aka zenith angle) up to about 60° (with respect to the normal of the front surface) but rather poor for high angles of incidence, as there is hardly any response for incidence angles above 80°. Increase in incidence angle lead to average angular responsivity gap widening (Fig. 2a), and relative angular responsivity drop between dosimeter and the ideal cosine (Fig. 2b). However, importantly, the angular responsivity does not depend on the azimuth of the light source (sun). Since the dosimeter is used to monitor personal exposure during a workday and worn somewhere on the human body, the dosimeter’s orientation in space is changing with body movements. It is impossible to correct the dosimeter measurements for the non-ideal (cosine) angular responsivity because the position of the sun with respect to the dosimeter surface is unknown. Similarly, the reflectivity of any (reflective) surface that might contribute to the radiation, falling on the dosimeter is unknown.

### Calibration

**Fig. 3:**
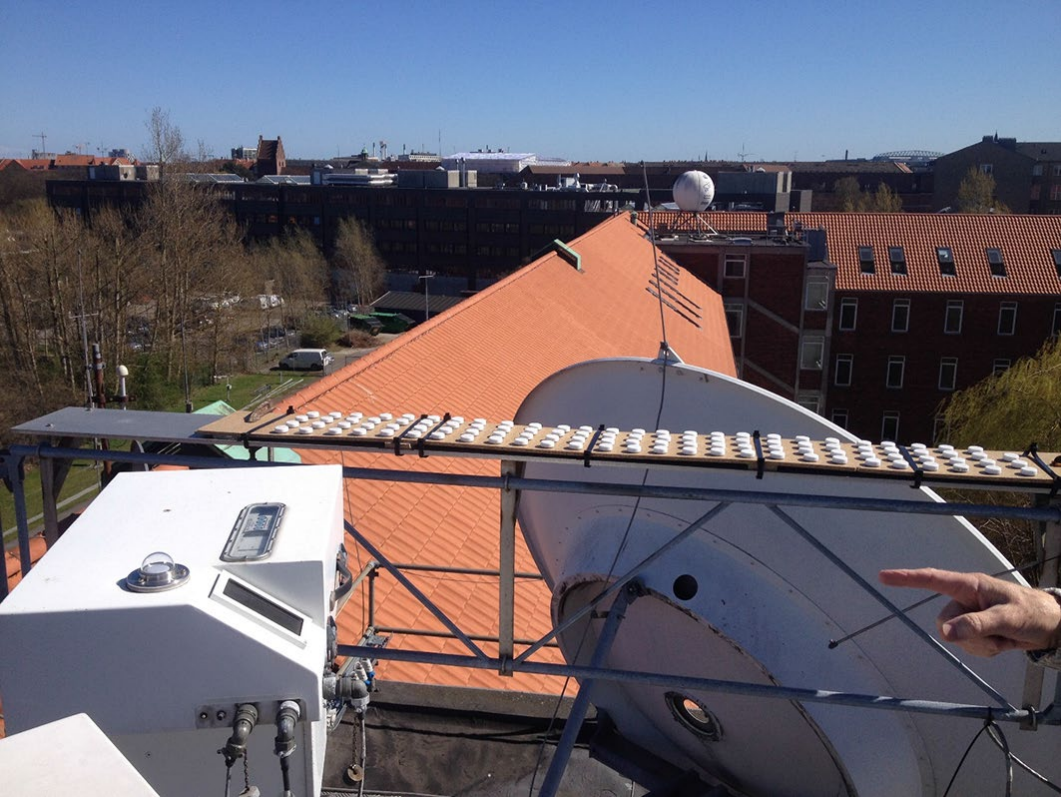
Calibration of dosimeters on 20 April 2016

On a cloudless day (20 April 2016), the dosimeters to be used in a field campaign during the summer of 2016 were placed horizontally and in close proximity to the Brewer MkIII spectrophotometer reference instrument on the roof of the Danish Meteorological Institute [9] (Fig. 3). Diffuse sky radiation coming from a very small solid angle to the north did not reach the dosimeters because of a shading structure. However, the angle of incidence of that radiation was in the region where the dosimeters’ angular responsivity was practically zero so the obstruction was not important. The dosimeters were exposed from dawn until about 2 PM, with measurements at 1-min intervals.

A calibration factor for each dosimeter, converting the dosimeters raw data to erythemally weighted irradiance, or standard erythema doses per hour (SED/h), was determined from a linear fit of each dosimeter’s raw data to the reference instrument’s erythemally (or CIE-) weighted irradiance. For reference, one SED equals an erythemal exposure of 100 J/m^2^ [8]. Because the dosimeters’ spectral responsivity is heavily weighted to the UV-B, the calibration factor depends on the ozone layer thickness on the day of calibration. Therefore, any subsequent dosimeter measurements should be corrected for the actual ozone layer thickness on the day of measurement. This correction can be calculated using the spectral responsivity (Fig. 1) and a radiative transfer model [10].

### Dosimeter deployment

The nationwide field campaign was set for the summer 2016. The dosimeters were sent to every participant’s home address using bubble wrap envelopes to prevent breakage and 1-day mail delivery services for timely delivery and returned the same way. On return the dosimeter’s data were read, the dosimeters were reset, cleaned, the battery changed and the straps were washed.

The participating workers wore the dosimeters either on a standard wristband or on a longer nylon strap (Buzz Rack), more compatible with outdoor workers’ clothes and gloves.

During the measurement period, we sent a daily reminder (text) to each participant’s mobile phone at 7:00 a.m. with instructions on how to use the dosimeter and when to use it. Likewise a message was sent to each participant at 7:00 p.m. with a yes–no question on whether the dosimeter was used as instructed and a question on the status of the particular workday.

**Fig. 4:**
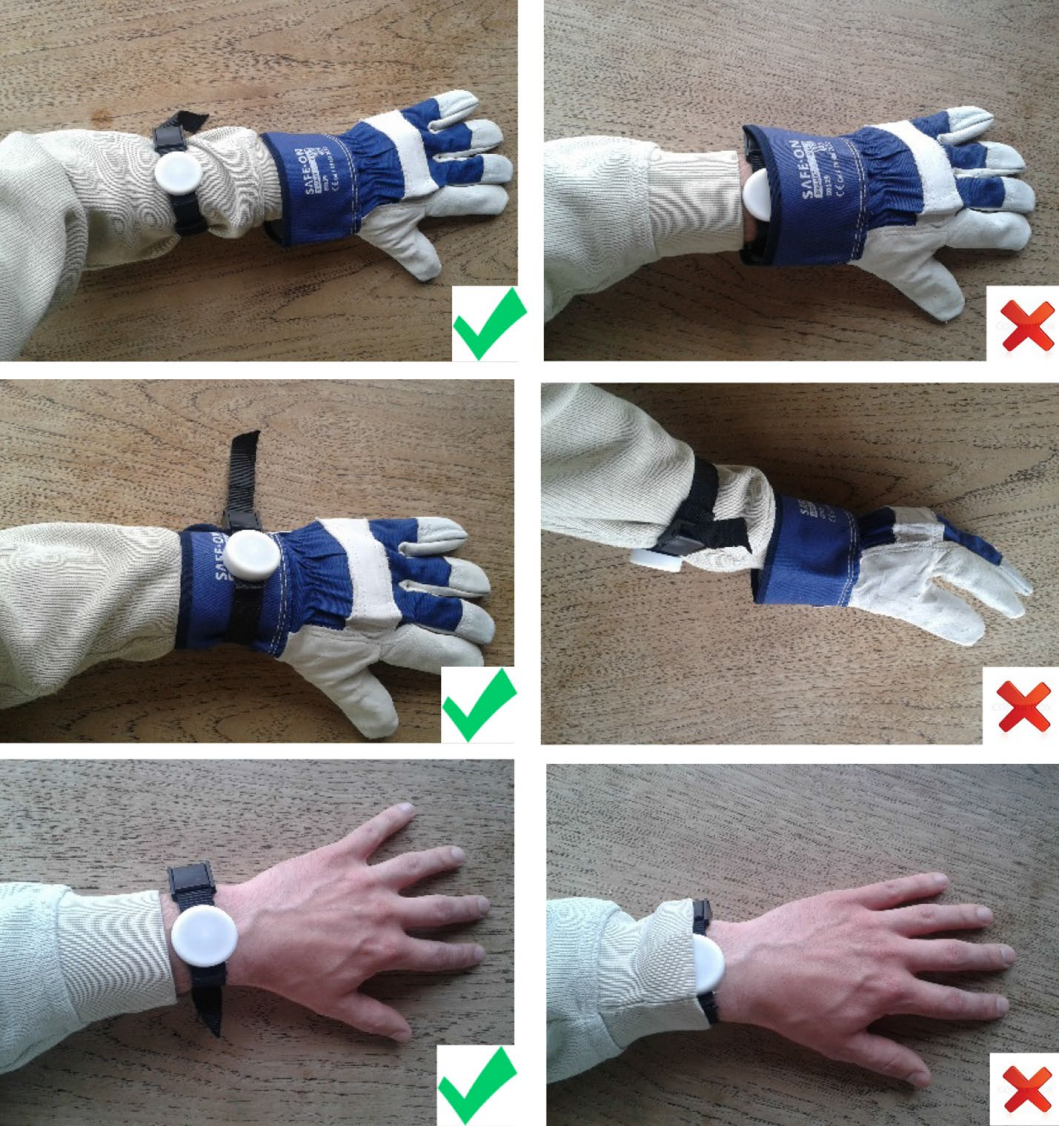
Visual instruction on how to wear the dosimeter

Participants were provided with timely oral, written and visual instructions in the use of the dosimeters to ensure anatomic uniformity (Fig. 4). In particular, the participants were instructed to wear the dosimeter on the dorsal side of the wrist or lower arm and to ensure that no fabric would cover the dosimeter.

### Anatomical location of dosimeters and measured exposure

The exposure (dose) measured depends on the dosimeter’s orientation with respect to the direction to the sun. We do not have measurements of the angular responsivity of the dosimeters utilized but we expect it to be much the same as that shown in Fig. 2 for an earlier version. As already mentioned it is impossible (with the capabilities of the current version of the dosimeter) to correct for non-ideal angular responsivity because the dosimeter changes orientation with arm movements. On average, the exposure measured on the dorsal side of the wrist equals half of the exposure measured on the scalp [11].

### Danish campaign measurements

The planned measurement period for each participant, was 2 weeks comprising ten workdays and 2 weekends, long enough to allow for different weather conditions. In practice, this was also achieved in average. Roughly, forty-five dosimeters were dispatched at 2-week intervals allowing for nine measurement cycles between mid-May and late September.

The dosimeters were pre-configured to take time-stamped measurements every 10 s from 7:00 a.m. until 7:00 p.m. local time, a high time resolution and a daily measurement period covering most of the daylight hours with local noon close to 1:00 p.m.

A similar field campaign is planned for 2017.

### Troubleshooting

There have been a few failures and shortcomings with the dosimeters during the campaign. First, Scienterra distributed a firmware update for the instruments in 2015. A fragment of old code persisted after the update was installed, which caused the instruments’ startup routine to become unstable. To an observer, the affected dosimeters appeared to have merely faulty batteries, and replacing the batteries could sometimes bypass the instability. However, during in-field measurements, there were periods of data loss, triggered when electrostatic discharges caused the instruments to reset and become unstable. During these periods, the timekeeping clock did not advance, which further compromised the measurement schedule. Loss of data during in-field application occurred in one of twenty-five cases in 2016. This is a higher loss compared to losses seen in other comparable studies and clearly not acceptable. Scienterra later addressed this problem with a firmware patch.

Furthermore, dosimeters were in risk of coming apart during use, particularly for participants performing manual labor. This occurred in one of seventy-five cases. However, our dosimeters were produced many years ago and we know that this design flaw has since been changed. For our dosimeters, this risk was minimized by slightly expanding the dosimeter-retaining ring for a firmer grip.

Finally, when using regular mail for sending back and forth the dosimeters to participants you risk losing dosimeters. This happened in about 1% of the cases. The lesson learnt is to send dosimeters with insured parcel post.

## Results and discussion

During the Danish campaign, we measured the personal UV exposure of more than three hundred and fifty participants employing one hundred and two dosimeters resulting in a total of about 4900 measurement-days altogether with 4321 data points each and roughly 60,000 data points per participant.

### Samples of exposure data from the campaign

**Fig. 5:**
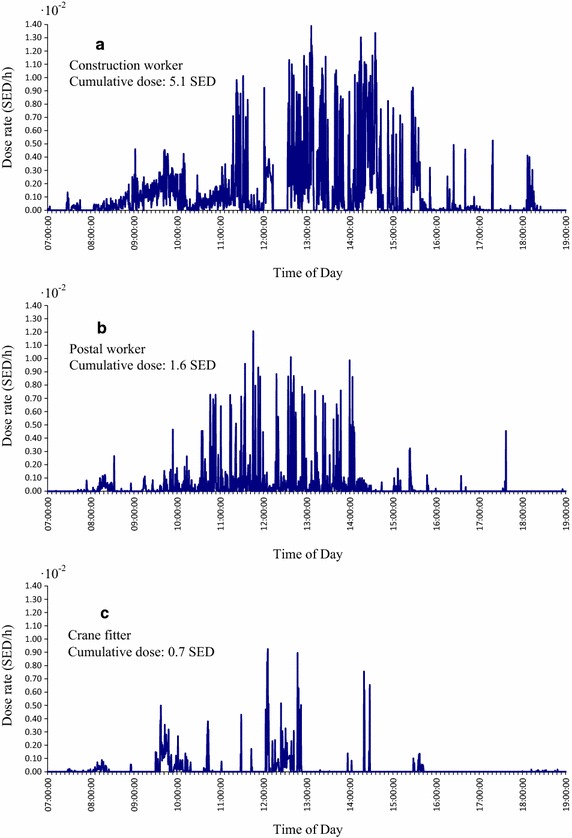
Three examples of occupational UV exposure measurements on the wrist on 2016-07-26, during the campaign. The ISO (International Standard Organization) 8-h threshold limit value is 0.3 SED (Standard Erythema Dose) set by the International Commission on Non-Ionizing Radiation Protection (ICNIRP) [12]

Here we present and discuss preliminary campaign occupational UV exposure data on 2016-07-26, a midsummer day with an average number of sun hours for three Danish outdoor workers; a construction worker with predominantly outdoor work, a postal service worker with intermittent outdoor work and a crane fitter with sparse outdoor work (Fig. 5).

This portrays a semi random selection of our data and illustrates the marked difference in UV exposure over time and accumulated daily dose between the included subjects representing different professions. The construction worker is exposed to high-levels of UV continuously for most of his working day, except during lunch hour (Fig. 5a). The postal worker is exposed to high-levels of UV intermittently between 10:00 a.m. and 14:00 p.m., but not exposed when mail packing forenoon (Fig. 5b). The crane fitter is only exposed to high-levels of UV during lunch hour (Fig. 5c). When comparing the three, the construction worker received thrice the accumulated SEDs of the postal worker, and seven times the accumulated SEDs of the crane fitter.

Given our specialist knowledge as occupational physicians, these findings are altogether as expected, and support the presumption that the Danish campaign UV-B dosimeter measurement dataset can be used to sum and compare exposure between groups of professions with reliable results to be used in future analysis with clinical as well as epidemiological/questionnaire data.
